# Free Flap Blood Flow Evaluated Using Two-Dimensional Laser Speckle Flowgraphy

**DOI:** 10.1155/2011/297251

**Published:** 2011-04-28

**Authors:** Toshiaki Furuta, Michihiko Sone, Yasushi Fujimoto, Shunjiro Yagi, Makoto Sugiura, Yuzuru Kamei, Hitoshi Fujii, Tsutomu Nakashima

**Affiliations:** ^1^Department of Otorhinolaryngology, Nagoya University Graduate School of Medicine, 65 Tsurumai-cho, Showa-ku, Nagoya 466-8550, Japan; ^2^Department of Otorhinolaryngology, Kariya Toyota General Hospital, 5-15 Sumiyoshi-cho, Kariya-city, Aichi 448-8505, Japan; ^3^Department of Plastic and Reconstructive Surgery, Nagoya University Graduate School of Medicine, 65 Tsurumai-cho, Showa-ku, Nagoya 466-8550, Japan; ^4^Department of Computer Science and Electronics, Kyushu Institute of Technology, 680-4 Kawazu Iizuka-city, Fukuoka 820-8502, Japan

## Abstract

*Objective*. We investigated the efficiency of laser speckle flowgraphy for evaluating blood flow in free flaps used for plastic surgery. *Methods*. We measured blood flow using a visual laser meter capable of providing two-dimensional color graphic representations of flow distribution for a given area using a dynamic laser speckle effect. Using laser speckle flowgraphy, we examined the blood flow of 20 free flaps applied following the excision of head and neck tumors. *Results*. After anastomosis of the feeding and draining blood vessels and sewing the flap, musculocutaneous (MC) flaps showed significantly lower blood flow than jejunal or omental flaps (*P* < .05). The ratio of blood flow decrease from the edge to the center was significantly greater in MC flaps than in jejunal or omental flaps (*P* < .001). *Conclusion*. Laser speckle flowgraphy is useful for the perioperative measurement of blood flow in free flaps used in plastic surgery. This method is a highly useful, practical, and reliable tool for assessing cutaneous blood flow and is expected to be applicable to several clinical fields.

## 1. Introduction

Microvascular free tissue transfer has become a common surgical technique in reconstruction of the head and neck. Its success rate is high but there are occasional failures. The success is influenced by the types of anticoagulation and radiation therapy used, and the existence of diabetes mellitus and arteriosclerosis in patients. Interoperative and postoperative monitoring of autologous tissue transplants is essential to identify complications early and minimize flap failure [1]. Many forms of monitoring have been developed over the last three decades, including measuring transcutaneous oxygen tension [2–4], differential tissue thermometry [5, 6], pedicle thermocouple probing [7], photoplethysmography [8, 9], ultrasonic Doppler flowmetry [10, 11], quantitative and qualitative fluorometry [12], evoked myoelectric waves [13], and laser Doppler flowmetry [14, 15].

Since the 1970s, methods for blood flow determination using a laser have been developed [16]. Laser Doppler measurement [17] led to the development of laser blood flowmeters, which are now widely used in clinical practice including the assessment and treatment of patients with laryngeal diseases [18]. Laser Doppler flowmetry is used most for the blood flow measurement of the flap at the peroperative period now. We have applied this type of flowmeter to the monitoring and assessment of free flap blood flow during plastic surgery. It provides a two-dimensional color graphic representation of peripheral circulation for a given area—a so-called blood flow map—through image sensor detection of dynamic laser speckles [19]. This method is called laser speckle flowgraphy, and the images obtained with this flow meter are called laser flowgrams. Laser speckle flowgraphy has the potential to create new applications utilising its higher resolution and faster response time, in addition to current Laser Doppler flowmetry [20]. Nakashima et al. [21] have applied this method to measure blood flow to the promontory in patients with cochlear otosclerosis. Laser speckle flowgraphy is used for the flow evaluation of retina, finger joint, and cerebrum during neurosurgery [22–24]. The present study aimed to investigate the efficacy of laser speckle flowgraphy for evaluating blood flow in free flaps used for tissue transfer during plastic surgery in patients treated for head and neck cancers.

## 2. Materials and Methods

### 2.1. Patients

The subjects were 20 patients (13 men and 7 women; mean age 60.7 years) with head and neck tumors that needed excision and microvascular free tissue flap transfers for repair.  Table 1 shows the clinical information for each case. Free jejunal flaps were used in 7 cases, omental flaps in 3 cases, and musculocutaneous (MC) flaps in 10. For the MC flaps, radial forearm flaps were used in 2 cases, transverse rectus abdominis musculocutaneous flaps in 3 and anterolateral thigh flap in 5. Six patients received preoperative radiation treatment; however, no patient had anticoagulation treatments.

### 2.2. Principle and Configuration of Laser Speckle Flowgraphy

**Figure 1:**
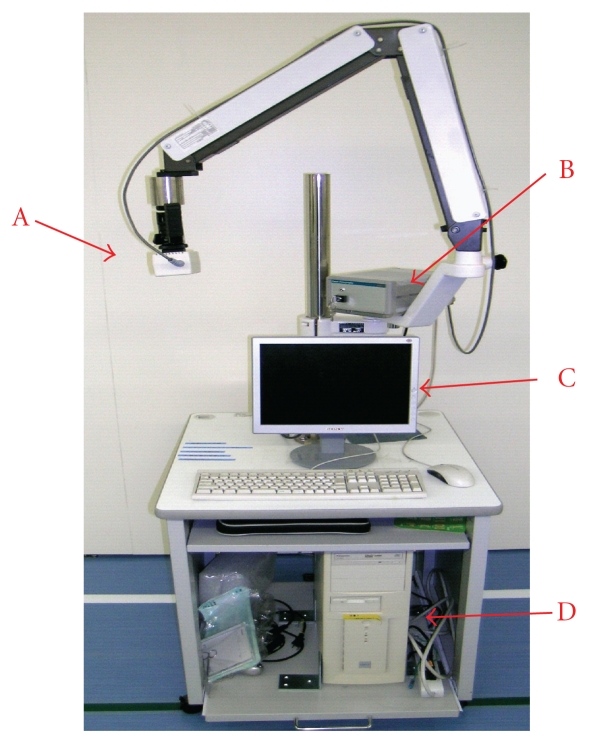
Photograph of the laser flowgraphy system. The laser probe is attached to the tip of the arm. (A) Device for discharging the laser and the mirror. (B) CCD (Charge Coupled Device) image sensor. (C) Monitor screen. (D) Computer used.

**Figure 2:**
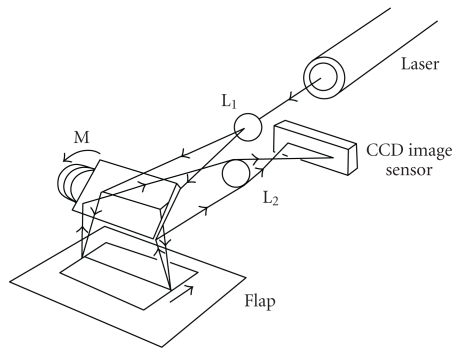
Schematic representation of visualization of two-dimensional blood flow distribution using laser speckle flowgraphy. (L_1_) Cylindrical lens. (M) Mirror. (L_2_) Lens 2.

The laser speckle flowgraphy system is shown in Figure 1. If a laser beam is aimed at the skin surface, dynamic laser speckles will form on the image sensor. These are caused by interference between reflected and scattered light within the skin. The speckles change according to the movement of blood cells in capillaries, and the speed of this movement reflects the blood flow rate. Using this principle, the laser beam is expanded into a linear ray by a cylindrical lens and projected by means of a mirror onto the skin surface as a linear spot as shown in Figure 2. The reflected, scattered laser beam is reflected again with the mirror and guided through the lens into the charge-coupled device (CCD) image sensor. The differences between two consecutive laser speckles are determined and averaged statistically for each picture element on the image sensor to yield a linear blood flow distribution. If the mirror is rotated perpendicularly, a two-dimensional blood flow map is obtained. A microcomputer was used for the control and calculations.

In the configuration used here, analysis of 100 × 100 picture elements using a 20-mV, 780-nm semiconductor laser and a high-sensitivity image sensor provided the blood flow distribution over an area of 75 × 65 mm in about 3 s [17]. In the color graphic laser flowgram, the area with highest blood flow is colored red and this changes to yellow and then blue as blood flow decreases, as indicated by the color code bar on the display. A numerical blood flow level in arbitrary units at any given point and a gradient of blood flow volume along a given vertical line in the observation field can be displayed on the screen routinely. The average derivative is a unit of blood flow measurement. The maximum and minimum average derivative values are 0 and 250. The laser probe is equipped with a 9-inch color display.

### 2.3. Blood Flow Measurement

**Figure 3:**
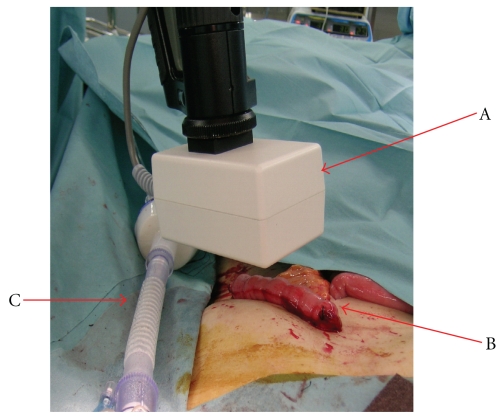
Photograph of measuring blood flow in the jejunal flap. (A) Device for discharging the laser and the mirror. (B) Jejunum. (C) Intubation tube.

Figure 3 shows a schematic of measuring blood flow in a free jejunal flap. It takes only about one minute to the measurement preparation of laser speckle flowgraphy. We measured the blood flow of the flap before the main nourishing vasculature had been separated and after the artery and the vein had been anastomosed and the flaps had been sutured. For surgery involving the mesopharynx and the nasal cavity, after the flaps had been sutured the laser could not be applied vertically. Therefore, it is not possible to measure blood flow accurately following placement, so it was measured during sewing.

**Figure 4:**
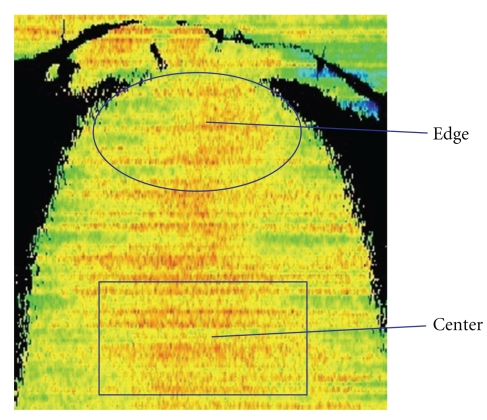
Laser flowgraphy in a case involving a musculocutaneous (MC) flap for treating a patient with tongue cancer.

In each case, the edges (10 mm inside of margin) and central portions of the flap were measured.  Figure 4 shows an example of a laser flowgraph in a case involving an MC flap for treating a patient with tongue cancer during separation of the main nourishing vasculature. The ratios of blood flow at the edge of flap to that in the center were calculated before and after vascular separation and after placement. During measuring, blood pressure was controlled to stabilize at 110–130 mmHg. Data were analyzed statistically using Stat Mate III software (http://www.atms.jp/). *P*-values <.05 were considered significant.

## 3. Results

The transplant of all flaps succeeded.  Table 2 shows average derivative values of the edges and flap centers before vascular separation and after sewing and matching the flap. When measuring the ratio of average derivative values after flap placement to average derivative values before main vessels were separated, MC flaps showed significant decreases in the center compared with jejunal (*P* < .03) and omental flaps (*P* < .03). MC flaps also showed significant decreases in the edges compared with jejunal (*P* < .01) and omental flaps (*P* < .01).

**Figure 5:**
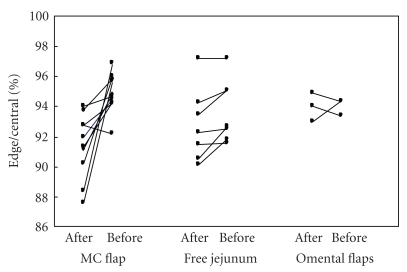
Blood flow ratios between the edge of flap to the flap center in a musculocutaneous (MC), jejunal and omental flaps, before vascular separation and after sewing the flaps.

Figure 5 shows the ratio of average derivative values in the edges to those in the centers of flaps in each group before vascular separation and after sewing and matching the flap. The ratio decreased significantly in MC flaps after they had been sewn compared with the ratio measured before the main vessels had been separated (*P* < .001). However, there were no significant differences in the ratios for jejunal or omental flaps. The blood flow measures did not show significant differences following radiation therapy.

## 4. Discussion

In general, arterial thrombi are found intraoperatively and venous thrombi are found postoperatively. In plastic and reconstructive surgery—where flap manipulation is essential—a tool for earlier and more detailed assessment of flap blood flow than can be achieved by macroscopic assessment is desirable clinically. 

The advantages of the laser speckle flowgraphy used currently are as follows.

1. It is noninvasive.
2. It can measure blood flow two-dimensionally in the area of interest within a short time.
3. It is easy to handle and interpret by junior physicians or nursing personnel.
4. It is highly transportable to the patient's bedside in the recovery room or the operating room.

**Figure 6:**
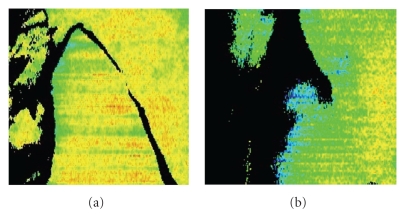
(a) Case that flow became bad when MC flap is collected. The MC flap on the other side was collected without the MC flap separating because the blood flow was bad. (b) State of flow on the second day of after the operation of the flap. The blood flow worsened and the edge of the flap got the sphacelation. The necrotic part of the flap is displayed blacking it.

The edge of a separated muscle flap loses blood flow easily. In MC flaps, the vascular structure is different from that in the jejunum or omentum. Our study showed a significant decrease of blood flow in the edge of MC flaps compared with jejunal and omental flaps. It is thought that the blood flow to the edge decreases still further when the flap is sewn in completely. In our preliminary experience, the low blood flow region presents as a blue area in which the flow level is approximately less than 80% of normal, indicating potentially necrotic tissue. The case is presented in Figure 6. When MC flap was collected, it was noticed that the blood flow of the flap was bad in laser speckle flowgraphy. MC flap on the other side was collected without the MC flap separating. In two days after the operation, the blood flow worsened and the edge of the flap got the sphacelation. The ratios of blood flow at the edge of flap to that in the center were 80%.

Based on our clinical use in plastic surgery, laser speckle flowgraphy allows recognition of a poorly perfused area of a flap during surgery, and resection of the distal flap area can be performed based on this finding. This flowmeter contributes greatly to the detection of changes in cutaneous blood flow in flaps or grafted tissue earlier than macroscopic observation. It is used additionally to ascertain macroscopic findings before selection of the next step of treatment. When the laser speckle flowgraph is used for postoperative followup of flap patency, minute changes in the microcirculation can also be observed, which cannot be detected macroscopically.

Mahabir et al. reported an inverse relationship between muscle vascular resistance and flap mass [25], indicating that larger muscles have less vascular resistance. This permits higher flow rates that could improve vein graft patency. The clinical implication is that a larger flap should be used when high flow-through is critical. We are planning to investigate this relationship between flap volume and blood flow using laser speckle flowgraphy.

It is known that thromboses in the microvascular anastomosis lead to failure in free tissue flap transfers. Technical and pharmacological advances have decreased the thrombogenic effect of abnormalities in blood vessels and endothelial lining and have helped decrease the coagulating propensity of blood during surgery. Equally important to patency of the microvascular anastomosis is blood flow, which is inversely proportional to the total resistance provided by the microcirculatory beds downstream. In this regard, we plan to characterize the vascular resistance, weight, volume and surface area of flaps available for transfer, and to identify favorable tissues for reconstruction from the standpoint of outflow resistance. Thus, laser speckle flowmetry is a highly useful, practical, and reliable tool for assessing cutaneous blood flow and is expected to be applicable to several clinical fields such as studying changes in blood flow during radiation therapy and changes in blood flow after surgery to the head and neck.

## 5. Conclusions

Laser speckle flowgraphy demonstrated a significant decrease in blood flow at the edge of MC flaps. This method is useful for evaluating blood flow in the microvasculature of free flaps used in plastic surgery.

## Figures and Tables

**Table 1:** The number of parts of cancer and kinds of flap.

Patient	Gender (female/male)	Ages	Primary sites	Flap	Radiation therapy (gray)
1	M	58	Oropharynx	MC Flap	—
2	M	64	Oropharynx	MC Flap	70
3	M	83	Oropharynx	MC Flap	—
4	M	84	Tongue	MC Flap	—
5	F	49	Tongue	MC Flap	—
6	F	48	Tongue	MC Flap	—
7	F	72	Tongue	MC Flap	30
8	M	60	Larynx	MC Flap	—
9	M	17	Nose/paranasal sinuses	MC Flap	—
10	F	72	Tongue	Mc Flap	—
11	F	54	Hypopharynx	Jejunum	40
12	M	59	Hypopharynx	Jejunum	—
13	M	76	Hypopharynx	Jejunum	60
14	M	54	Hypopharynx	Jejunum	64
15	F	50	Hypopharynx	Jejunum	40
16	M	67	Hypopharynx	Jejunum	—
17	M	70	Hypopharynx	Jejunum	—
18	M	70	Parotid gland	Omental	—
19	M	69	Parotid gland	Omental	—
20	F	28	Nose/paranasal sinuses	Omental	—

MC Flap: musculocutaneous flaps. M: male. F: female.

**Table 2:** Average derivative of edge and the flap center at the previous state of main nourishment vascular separation before and sewing the flap after.

Patient	Flap	Center after/before (ratio %)	Edge after/before (ratio %)
1	MC Flap	167/184 (90.8)	154/174 (88.5)
2	MC Flap	149/172 (86.6)	140/165 (84.8)
3	MC Flap	139/163 (85.3)	123/159 (77.4)
4	MC Flap	173/176 (98.3)	158/169 (93.5)
5	MC Flap	162/177 (91.5)	148/167 (88.6)
6	MC Flap	167/171 (97.5)	157/162 (96.9)
7	MC Flap	138/168 (82.1)	131/154 (85.1)
8	MC Flap	165/207 (79.7)	153/191 (80.1)
9	MC Flap	185/194 (95.4)	170/177 (96.0)
10	MC Flap	170/173 (98.3)	155/164 (94.5)
11	Jejunum	78/80 (97.5)	72/74 (97.3)
12	Jejunum	88/90 (97.8)	82/86 (95.3)
13	Jejunum	81/86 (94.2)	73/79 (92.4)
14	Jejunum	53/55 (96.4)	48/51 (94.1)
15	Jejunum	71/72 (98.6)	65/66 (98.5)
16	Jejunum	72/73 (98.6)	70/71 (98.6)
17	Jejunum	78/82 (95.1)	73/77 (94.8)
18	Omental	151/152 (99.3)	142/142 (100)
19	Omental	157/159 (98.7)	149/150 (99.3)
20	Omental	157/159 (98.7)	149/150 (97.3)

After: after sewing and matching the flap. Before: before vascular separation. MC Flap: musculocutaneous flap.
